# The Ubiquitin-Proteasome Reporter GFPu Does Not Accumulate in Neurons of the R6/2 Transgenic Mouse Model of Huntington's Disease

**DOI:** 10.1371/journal.pone.0005128

**Published:** 2009-04-08

**Authors:** John S. Bett, Casey Cook, Leonard Petrucelli, Gillian P. Bates

**Affiliations:** 1 King's College London School of Medicine, Department of Medical and Molecular Genetics, King's College London, London, United Kingdom; 2 Mayo Clinic, Jacksonville, Florida, United States of America; University of Giessen, Germany

## Abstract

Impairment of the ubiquitin-proteasome system (UPS) has long been considered an attractive hypothesis to explain the selective dysfunction and death of neurons in polyglutamine disorders such as Huntington's disease (HD). The fact that inclusion bodies in HD mouse models and patient brains are rich in ubiquitin and proteasome components suggests that the UPS may be hindered directly or indirectly by inclusion bodies or their misfolded monomeric or oligomeric precursors. However, studies into UPS function in various polyglutamine disease models have yielded conflicting results, suggesting mutant polyglutamine tracts may exert different effects on the UPS depending on protein context, expression level, subcellular localisation and cell-type. To investigate UPS function in a well-characterised mouse model of HD, we have crossed R6/2 HD mice with transgenic UPS reporter mice expressing the GFPu construct. The GFPu construct comprises GFP fused to a constitutive degradation signal (CL-1) that promotes its rapid degradation under conditions of a healthy UPS. Using a combination of immunoblot analysis, fluorescence and immunofluorescence microscopy studies, we found that steady-state GFPu levels were not detectably different between R6/2 and non-R6/2 brain. We observed no correlation between inclusion body formation and GFPu accumulation, suggesting no direct relationship between protein aggregation and global UPS inhibition in R6/2 mice. These findings suggest that while certain branches of the UPS can be impaired by mutant polyglutamine proteins, such proteins do not necessarily cause total blockade of UPS-dependent degradation. It is therefore likely that the relationship between mutant polyglutamine proteins and the UPS is more complex than originally anticipated.

## Introduction

Huntington's disease (HD) is a neurodegenerative disorder caused by the expansion of a polyglutamine tract in the N-terminus of the 348 kDa protein huntingtin (htt). It is one of a family of diseases caused by a polyglutamine expansion, and is characterised by the misfolding, aggregation and deposition of polyglutamine-expanded N-terminal htt into intracellular inclusion bodies [1]. While mutant htt has been proposed to exert its toxicity through various mechanisms including transcriptional dysregulation [2] and disturbances to protein folding networks [3], the finding that htt inclusion bodies are polyubiquitylated in transgenic mice and HD patient brains has long suggested that altered ubiquitin homeostasis or impaired ubiquitin-proteasome system (UPS)-dependent protein degradation may contribute to HD pathology [1], [4].

The UPS is an essential cellular mechanism responsible for the timely degradation of both healthy and damaged or misfolded proteins. Degradation by the UPS requires that a protein is first tagged with a minimum of four Lys48-linked ubiquitin monomers before shuttling to and recognition by the 26S proteasome. The 26S proteasome is a multi-subunit and multi-catalytic machine which unfolds, deubiquitylates, and digests its substrates into short peptide fragments in an ATP-dependent manner [5]. Because of its fundamental requirement to cellular viability, inhibition of any of these steps as a result of protein aggregation or the inability to handle specific toxic proteins could be responsible for the death and dysfunction of neurons in HD and other polyglutamine/protein conformation disorders [6].

It is becoming clear that disturbed ubiquitin homeostasis is closely linked with HD pathology, as accumulation of polyubiquitin chains and increased levels of monoubiquitylated histone H2A (uH2A) have been reported in HD mouse tissues [7]–[9]. It is still currently unclear if mutant polyglutamine proteins cause a general impairment of the UPS however. Assays of proteasome activity using fluorogenic peptides exhibit normal or increased proteasomal activity in brain extracts of various polyglutamine disease mouse models [10]–[12], although human post-mortem HD brains have shown diminished core proteasome activity [13]. In support of a general blockade of UPS-dependent protein degradation, it has been shown that the presence of a mutant polyglutamine tract can hinder a protein's proteasomal degradation [14], [15], leaving open the possibility that long polyglutamine stretches may inactivate the 26S proteasome by becoming trapped in the proteolytic chamber [16]. Adding credence to this hypothesis was the report that eukaryotic proteasomes are unable to degrade polyglutamine tracts [17]. However, recent data demonstrates that polyglutamine tracts are degraded efficiently by eukaryotic proteasomes, thereby refuting the proposal that mutant polyglutamines block 26S proteasome function by becoming trapped inside the 26S proteasome [18].

Biochemical assays have been very useful in rapidly assessing the status of both 20S and 26S proteasome activity in polyglutamine-disease tissue extracts by measuring degradation of non-ubiquitylated fluorogenic substrates and ubiquitylated-lysozyme substrates respectively [10]–[13]. However, these assays do not involve substrate passage through all steps of physiologically relevant UPS-dependent protein degradation pathways. An alternative has been the use of recombinant probes typically comprised of enhanced green fluorescent protein (GFP) appended with a destabilising modification which promotes their constitutive degradation by the UPS [19], [20]. Degradation signals utilised have included the ubiquitin-fusion degradation (UFD) signal [21], [22], where an uncleavable N-terminal ubiquitin fusion directs the protein to the UPS; the N-end rule signal [21], where certain N-terminal amino acids cause rapid UPS-mediated protein turnover; and the CL-1 degron [23], a destabilising C-terminal 16 amino acid sequence used to generate the “GFPu” UPS reporter construct. The CL1 degron was originally identified in a yeast screen for peptides that destabilise proteins in a manner dependent on the E2 ubiquitin-conjugating enzymes Ubc6 and Ubc7, but other E2s are also believed to promote CL-1 degradation in mammalian cells (reviewed in [19]). GFPu has previously been shown to co-immunoprecipitate with ubiquitin and accumulate when the proteasome is inhibited, supporting its validity as a reliable UPS reporter [23]. Although each different class of reporter requires different combinations of ubiquitin conjugating (E2) and ligating (E3) enzymes and exhibit distinct stabilities, they are all believed to converge on the same pool of 26S proteasomes. Assuming that the rates of synthesis of these reporters are not perturbed, their steady-state levels in a given cell type reflects overall flux through the UPS.

The first direct evidence that an expanded polyglutamine protein can impair the UPS came from *in vitro* studies showing mutant htt aggregation causes the accumulation of a stably-expressed GFPu UPS reporter [23]. While similar observations have been observed in cell models of other polyglutamine diseases [24]–[26], no impairment was observed in a polyglutamine-expanded ataxin-1 cell model using UFD or N-end rule UPS reporters [27]. Impairment of the UPS has been observed *in vivo* in a *C.elegans* spinocerebellar ataxia 3 (SCA3) model using a GFPu UPS reporter [28], while contrasting evidence from an SCA7 mouse model shows that polyglutamine pathogenesis can occur in the absence of significant UPS impairment, as detected by a UFD reporter [29]. More recently, UPS impairment was observed in synapses of transgenic R6/2 HD mouse neurons upon injection of viral vectors harbouring the GFPu reporter [30]. Interestingly, GFPu accumulation did not occur in neuronal cell bodies in this study, suggesting that while subcellular localisation can affect UPS efficiency in polyglutamine disease, mutant polyglutamine proteins do not necessarily cause general impairment of the UPS.

In the current study, we have crossed transgenic R6/2 HD mice [31] with transgenic GFPu mice [32] to investigate potential impairment of the UPS in a well-established mouse model of HD. We found that GFPu protein levels are unchanged in R6/2 whole brain extracts, and that there is no accumulation of GFPu in R6/2 neurons relative to inclusion body formation. This suggests that while mutant htt can cause disturbances to ubiquitin homeostasis and in some cases impaired degradation of UPS substrates, it may not necessarily cause a general blockade of UPS-dependent protein degradation.

## Results

### Genetic cross of R6/2 transgenic HD mice with GFPu UPS reporter mice

**Figure 1:**
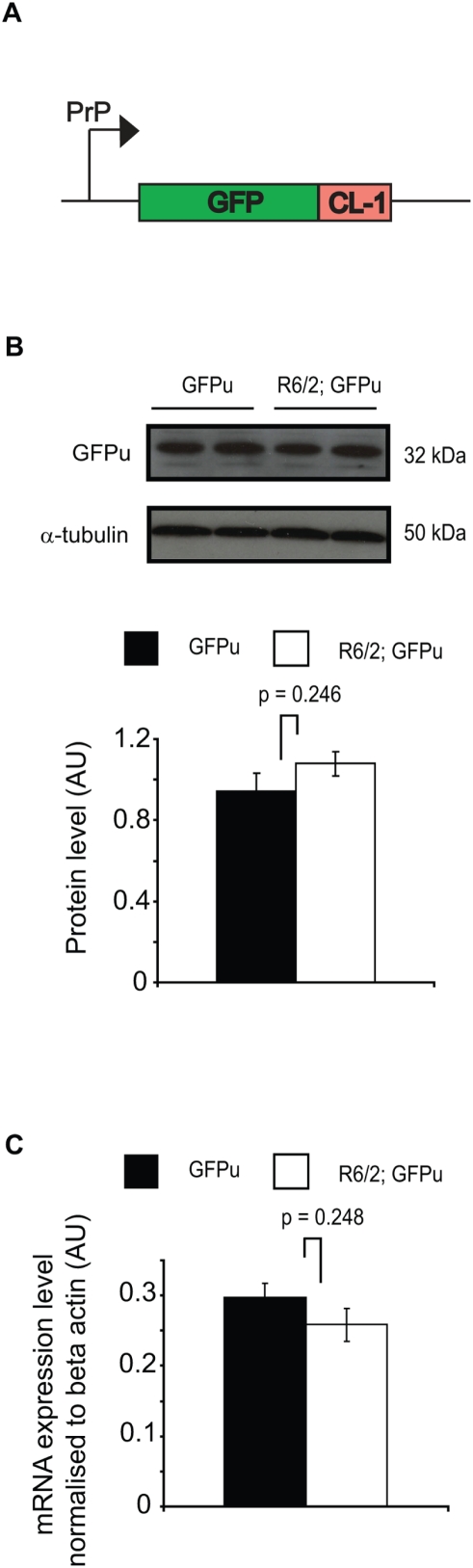
GFPu does not accumulate in the R6/2 brain. (A) Schematic showing the GFPu construct under control of the mouse prion promoter (PrP). GFPu protein is composed of GFP appended with a 16 amino acid C-terminal degradation signal, the CL-1 degron. (B) Western blot analysis and densitomeric quantification reveals no increase in steady-state levels of GFPu in 12 week R6/2 brains. α-tubulin was used as a loading control. (C) Expression of the GFPu transgene is unchanged in the 12-week-old R6/2 brain. Error bars represent the standard error of the mean.

To test the hypothesis that mutant htt expression causes general impairment of the UPS *in vivo*, we crossed the well-characterised R6/2 mouse model of HD [31] with GFPu UPS reporter mice [32], which express the GFPu construct under the control of the mouse prion promoter (Figure 1A). R6/2 mice express exon 1 of htt harbouring a polyglutamine tract over 150 residues, and develop a progressive neurological phenotype from around 4–5 weeks of age. GFPu mice express the UPS reporter construct GFPu under the control of the mouse prion promoter, which accumulates in cultured mouse neurons under conditions of proteasome impairment [32]. GFPu males were crossed with 5-week-old R6/2 females to generate progeny of four genotypes to be used in subsequent experiments: wild type (WT), R6/2, GFPu and R6/2; GFPu. Mouse brains were harvested for analysis at 12 weeks, at which point R6/2 mice are at an advanced stage of disease.

If general impairment of the UPS can be caused by the expression of pathogenic polyglutamine proteins *in vivo*, then the GFPu fusion protein should accumulate in the brains of R6/2; GFPu mice. To investigate this possibility, GFPu levels were compared between 12-week-old GFPu mice and double-transgenic R6/2; GFPu mice. Western blot analysis of four whole-brain lysates per genotype followed by densitometric quantification revealed that the levels of GFPu were not altered in R6/2 mice (Figure 1B), in agreement with lack of gross proteasome inhibition in end-stage R6/2 brains [12]. To ensure that detection of impairment of the UPS in R6/2 brains was not obscured by dysregulated expression of the GFPu transgene, quantitative real-time RT-PCR analysis was carried out to monitor expression of GFPu mRNA. A GFP real-time expression assay was designed and expression levels of GFPu were compared between four brains of each genotype (GFPu and R6/2; GFPu). It was found that GFPu mRNA expression was unchanged in R6/2; GFPu double-transgenic mice (Figure 1C), suggesting the lack of difference in GFPu protein levels in R6/2 mice is not confounded by altered levels of GFPu mRNA.

### Levels of native GFP fluorescence are unchanged in R6/2; GFPu mice

**Figure 2:**
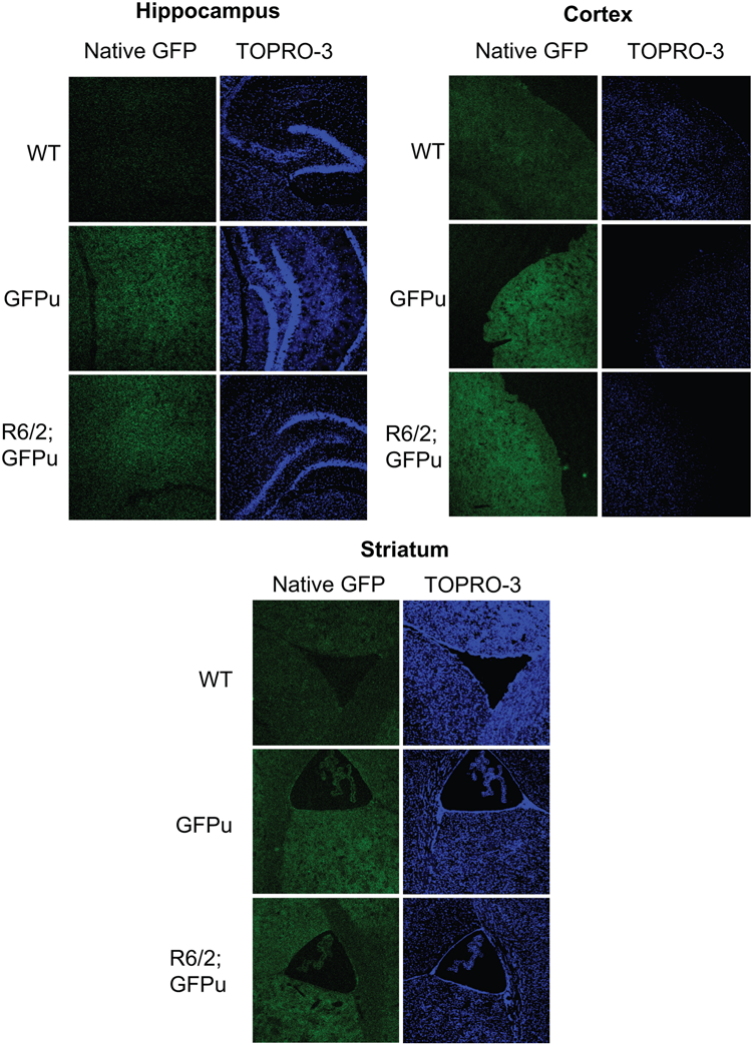
Native GFPu fluorescence in R6/2-GFPu mice. Native GFPu fluorescence is not notably increased in the hippocampus, cortex or striatum of R6/2; GFPu mice. Sections were stained with the nuclear-specific fluorescent dye TOPRO-3. Scale bars are 40 µM.

Although western blot analysis of R6/2-GFPu mice suggested that overall UPS function was normal in the R6/2 brain, it remained possible that the UPS was impaired in specific brain regions. To investigate this, sections of various gross brain regions from 12-week-old R6/2-GFPu mice were analysed by confocal microscopy. Sections were prepared side-by-side and confocal settings were unchanged after initial correction for background fluorescence in WT brains. Imaging of GFPu in the striatum, cortex and hippocampus from four brains per genotype revealed very comparable fluorescence levels between GFPu and R6/2; GFPu (Figure 2), suggesting normal processing of GFPu in these regions of the R6/2 brain. GFP fluorescence was widespread in these regions suggesting that the GFPu fusion protein is present throughout brain cells and neuronal processes, and the relatively low level of native GFP fluorescence is likely due to the rapid turnover of GFPu by the UPS. The GFPu protein appears to be equally expressed in both neurons and glia. This was unexpected given that the prion promoter drives higher expression in neurons, and suggests that transgene expression may have been influenced by effects at the site of integration.

### GFPu immunofluorescent intensity is unaltered in the R6/2 brain

**Figure 3:**
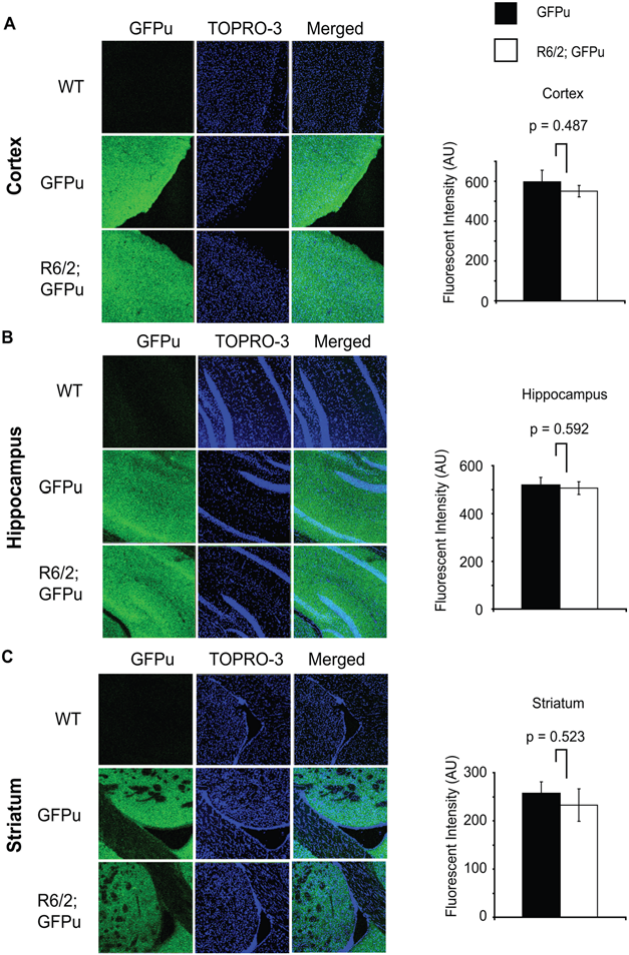
GFPu immunofluorescence in R6/2-GFPu mice. Immunofluorescent staining of brain sections with anti-GFP antibody followed by quantitation of fluorescent units reveals no difference in the levels of GFPu in R6/2 cortex (A), hippocampus (B) or striatum (C). Sections were counterstained with the nuclear fluorescent dye TOPRO-3. Error bars represent the standard error of the mean. Scale bars are 40 µM.

To confirm the results of the native GFPu imaging and to quantify levels of GFPu, 12-week-old brain sections from R6/2-GFPu mice were stained with an anti-GFP antibody and fluorescently labelled secondary antibody. Preliminary experiments confirmed that sections prepared for antibody staining did not emit any native GFP fluorescence (data not shown). Images captured from five brains per genotype suggested that there was no difference in the amount of GFPu immunofluorescent staining in the cortex, hippocampus or striatum between GFPu and R6/2; GFPu mice, confirming that GFPu does not accumulate in these regions in the R6/2 brain (Figure 3). To confirm that there was no difference in GFPu levels in the R6/2 brain, fluorescent intensity was carefully measured in each of the brain regions examined. Fluorescent intensity in randomly selected areas of the striatum, cortex and hippocampal neuronal layers was not significantly different between GFPu and R6/2; GFPu genotypes, suggesting an absence of general UPS impairment in the R6/2 brain (Figure 3).

### GFPu does not accumulate in neurons with inclusion bodies

**Figure 4:**
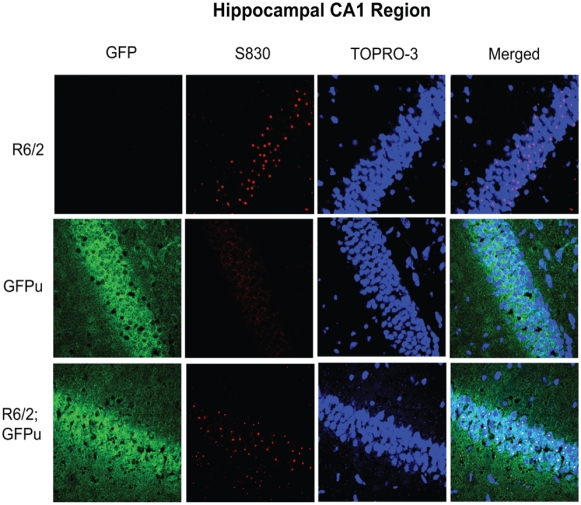
GFPu immunofluorescence and inclusion body formation in the hippocampus. GFPu immunofluorescence in the CA1 region of the hippocampus is comparable in 12-week-old GFPu and R6/2; GFPu brains. Staining with anti-htt antibody S830 shows widespread inclusion body formation in R6/2 mice. Sections were stained with the nuclear fluorescent dye TOPRO-3. Scale bars are 10 µM.

**Figure 5:**
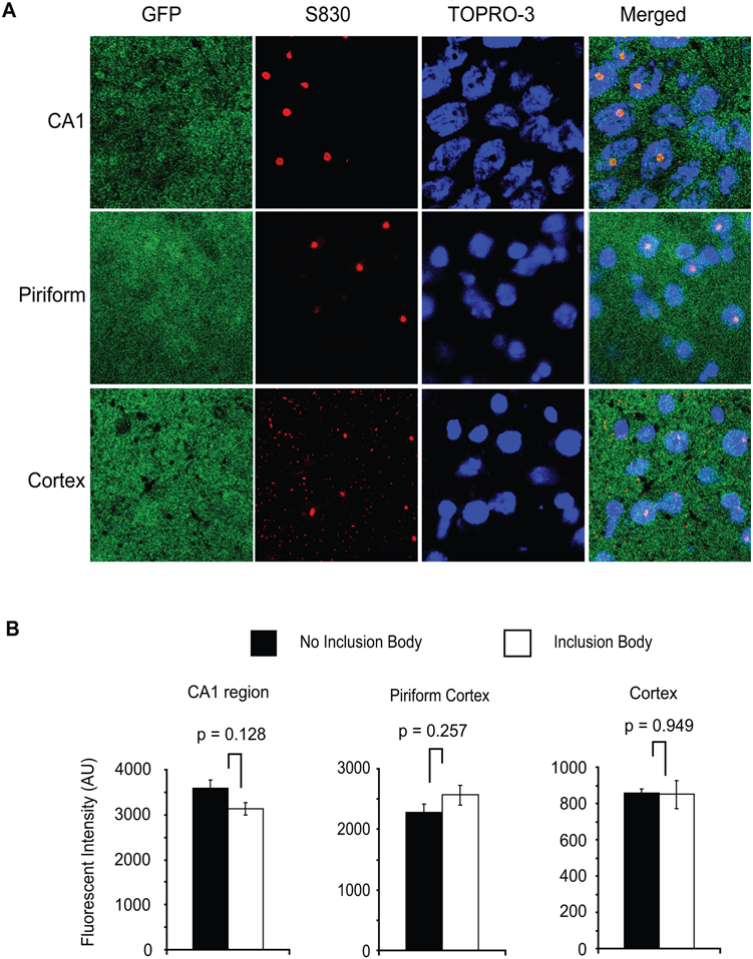
Relationship between the UPS and inclusion formation. (A) Immunofluorescent double-staining of R6/2; GFPu brain sections shows that the presence of nuclear inclusion bodies does not correlate with the intensity of GFPu immunofluorescence in CA1 region of the hippocampus, the piriform cortex or the cortex (B). Quantification of GFPu immunofluorescence in nuclei with or without an inclusion body confirms that there is no relationship between inclusion body formation and impairment of the UPS in R6/2 mice. Sections were stained with the nuclear fluorescent dye TOPRO-3. Error bars represent the standard errors of the mean. Scale bars are 6 µM.

In contrast to native GFPu imaging, immunofluorescent staining revealed a high concentration of GFPu in the densely-packed neuronal layers of the hippocampus, including the dentate gyrus and CA1 region, suggesting immunofluorescent staining may be more sensitive to variations in GFPu levels than native GFP fluorescence. To further investigate the possibility that GFPu accumulates in neurons of the hippocampal CA1 region, and to investigate any relationship between the UPS and inclusion body formation, immunofluorescent double-staining of hippocampal sections was performed with both anti-GFP and anti-htt antibody S830. High magnification images revealed that GFPu levels were similar between GFPu and R6/2; GFPu mice in the CA1 region, despite widespread inclusion body formation in the R6/2 brain (Figure 4). Although there was no gross accumulation of neuronal GFPu in the R6/2 brain, it was important to determine whether there was any relationship between inclusion body formation and GFPu concentration. To this end, GFPu immunofluorescence was compared between nuclei with or without an inclusion body in three distinct brain regions. Visual examination of the images suggested that there was no increase in GFPu concentration in nuclei containing an inclusion body in the CA1 region of the hippocampus, piriform cortex or cortex (Figure 5A). Quantification of fluorescent intensity in at least four nuclei with or without an inclusion body confirmed that the levels of GFPu did not correlate with inclusion body formation in any of these regions in the R6/2 brain (Figure 5B). Therefore, we have failed to detect a relationship between the aggregation of mutant htt and the efficiency of the UPS *in vivo*.

## Discussion

In this study, we have set out to investigate UPS function in the R6/2 mouse model of HD, using the degradation of the artificial UPS substrate GFPu as a reporter of *in vivo* UPS activity. We found by immunoblot analysis that steady-state levels of GFPu were unchanged in the R6/2 brain, in keeping with the normal extractable *in vitro* degradative capacity of proteasomes [12]. In agreement, immunofluorescence studies demonstrated that there is no net accumulation of GFPu in different regions of the R6/2 brain. The pyramidal neurons of the hippocampal CA1 region were examined at high magnification for any change in GFPu immunofluorescence levels in R6/2 mice. However, GFPu levels were not significantly different in R6/2 mice despite widespread inclusion body formation. Further analysis suggested that there was no correlation between GFPu levels and inclusion body formation in R6/2 neurons.

Notwithstanding any limitations of GFPu as a sensitive reporter of UPS function therefore, we have failed to detect a global inhibition of the UPS in R6/2 HD mice. As a surrogate measure of UPS activity however, it is possible that the expression of GFPu in mouse neurons could be affected such that its steady-state levels are not an accurate representation of global UPS activity. For example, the synthesis of GFPu protein may be delayed as a result of polyglutamine pathogenesis or proteins may associate with the CL-1 degron differently between WT and R6/2 neurons, thereby differentially affecting GFPu stabilisation. However, previous work with cultured cortical neurons from GFPu mice showed that GFP fluorescence accumulated in response to proteasome inhibition in a dose-dependent manner [32], and stereotaxic injection of the proteasome inhibitor MG-132 to GFPu mouse brains also lead to GFPu accumulation (manuscript in preparation). This suggests that *in vivo*, steady-state levels of GFPu are likely to act as a reliable indicator of global UPS function. In addition, our study is in agreement with recent studies by Wang et al., who showed that while UPS function may be compromised in R6/2 synapses, the GFPu fusion protein was degraded normally in the cell body [30]. This observed impairment of the UPS in R6/2 cell synapses [30] coupled with the finding that K48-linked polyubiquitin chains accumulate in R6/2, *Hdh* ^Q150/Q150^ HD knock-in mice and HD patient brains [7] suggests that while UPS impairment can occur as a consequence of mutant htt expression, it is not necessarily a global consequence of mutant htt expression. Although we cannot rule out the possibility that we have failed to detect general UPS impairment due to technical limitations of our GFPu measurements, the current study is in agreement with another that failed to detect UPS impairment in an SCA7 model using a UFD-based GFP reporter [29], a reporter that successfully detected UPS impairment in prion-infected mice [33].

While it is clear that the UPS is an important cellular defence against toxic polyglutamine protein expression [34], it is less clear how its efficiency can be hindered by expression of mutant polyglutamine proteins. Early hypotheses suggested mutant polyglutamine proteins could inhibit the 26S proteasome through undegradable polyglutamine stretches becoming trapped inside the proteasome proteolytic chamber [16]. In favour of this, it has been shown that polyglutamine proteins are intrinsically difficult to degrade [14], [15] and it has been reported that eukaryotic proteasomes are unable to degrade within polyglutamine tracts [17]. However, conflicting results have shown that polyglutamine tracts are cleaved at multiple sites by eukaryotic proteasomes [18], and targeting mutant polyglutamine proteins to the UPS is sufficient for their degradation [35], [36]. In addition, the fact that extractable 26S proteasome activity is normal in R6/2 brains [12] and the current finding that GFPu steady-state levels are unchanged in R6/2 neurons suggests that any impairment of the UPS caused by mutant polyglutamines does not occur through direct blockage of the 26S proteasome. This is supported by the *in vitro* finding that impairment of the UPS can occur in the nucleus and cytoplasm irrespective of the compartment to which mutant htt is restricted [25]. Any impairment of the UPS which may occur in for example, the synapse, may in fact be secondary to local reductions in ATP or axonal transport defects [30]. However, it has also been shown that while inclusion bodies themselves do not impair UPS function [25], filamentous aggregates purified from HD brains are capable of impairing the 26S proteasome *in vitro* [37].

The roots of the discrepancies in UPS efficiency observed between different model polyglutamine-disease systems are unclear. Using fluorescent UPS reporters, impairment of the UPS has been observed in some cell models [23], [24], [26] but not others [27]. In addition, global UPS impairment has been reported in a *C.elegans* polyglutamine disease model [28] but not in mouse models [29]. It is possible therefore, that mutant polyglutamine proteins do not necessarily cause a global impairment of the UPS, but rather may affect certain branches of the UPS depending on expression levels, cell type and subcellular localisation. The growing evidence for the involvement of UPS impairment in other neurodegenerative protein conformation disorders such as prion and Parkinson's disease [33], [38] may not therefore be directly applicable to the polyglutamine diseases. However, disturbances in ubiquitin homeostasis are emerging as being closely linked to polyglutamine disease progression [7], [9]. In addition to proteolysis, protein ubiquitylation affects a diverse range of cellular processes such as transcription, DNA repair, endocytosis, so perturbations in the ubiquitin system are likely to have global cellular consequences. It is very likely that elucidating the role of disturbed ubiquitin homeostasis in HD and the polyglutamine diseases will be informative with respect to unravelling the molecular pathology of these disorders.

## Materials and Methods

### Mouse husbandry

Mice were housed and experimental procedures performed in accordance with Home Office regulations. Animals had unlimited access to water and number 3 rodent breeding chow (Special Diet Services, Witham, UK), and were subject to a 12 h light (08:00–20:00), 12 h dark (20:00–08:00) cycle. Mice were housed 5 to a cage with environmental enrichment in the form of paper shred bedding (Enviro-dri, Lillico, Betchworth, UK), a play tunnel (Datesand Ltd., Manchester, UK) and wood shavings (GLP Aspen Chips, Datesand Ltd., Manchester, UK). R6/2 transgenic HD mice were generated as described previously [31]. The R6/2 mouse colony was maintained by backcrossing R6/2 males to (C57BL/6JxCBA/Ca) F1 females (B6CBAF1/OlaHsd, Harlan Olac, Bichester, UK). GFPu mice were generated as previously described [32] and maintained on a C57BL/6J background (Harlan Olac, Bichester, UK).

### Western blot analysis

Mice were culled by cervical dislocation and brains snap frozen in isopropanol on dry ice. Brain hemispheres were homogenised in sodium phosphate buffer [20 mM sodium phosphate pH 7.4, 1% SDS and complete protease inhibitors (Boehringer Mannheim)] and sonicated for 30 s. Protein concentration was measured using the BCA (bicinchoninic acid) assay kit (Pierce) and 20 µg protein was loaded onto 10% SDS-polyacrylamide gels before transfer to nitrocellulose membrane for immunoblotting. Following transfer, membranes were rinsed in PBS and then incubated for 1 h in 5% non-fat dry milk (NFDM) in PBS on a shaker. Membranes were probed with anti-GFP antibody (Abcam, 1∶1000) in 5% NFDM for 2 h at room temperature then rinsed four times in PBS on a shaker for 5 min per wash before incubation with horse radish peroxidase (HRP)-linked anti-rabbit secondary (Amersham, 1∶3000) in 5% NFDM. Membranes were then washed four times in PBS and protein was detected using the Enhanced Chemiluminescence (ECL) kit and Hyperfilm ECL (Amersham). Membranes were stripped in stripping buffer (100 mM β-mercaptoethanol, 2% SDS, 62.5 mM and Tris.HCl pH 6.7) at 50°C for 20 min with occasional agitation and reprobed with anti-α-tubulin (Sigma, 1∶2000) and HRP-linked anti mouse secondary (Vectastain, 1∶5000). ECL immunoblot signals were quantified on a Bio-Rad GS-800 Calibrated Densitometer using Quantity-One® software. Protein levels of four brains per genotype were quantified in three independent experiments and Student's t-test was performed to compare protein levels between genotypes.

### Quantitative real-time RT-PCR analysis

Mice were sacrificed by cervical dislocation and brains were quickly dissected, frozen in isopentane on dry ice and stored at −80°C until required. Total RNA was extracted from whole brain using the RNeasy lipid mini kit (Qiagen). Quality and quantity of RNA was assessed using the RNA nanochip method on a BioAnalyzer according to the manufacturer's instructions (Agilent Technologies). Reverse transcription of total brain RNA into single-stranded cDNA was carried out in two steps. First, 1 µg RNA was incubated with 100 ng random hexamer nucleotides and 100 mM DTT in diethylpyrocarbonate (DEPC)-treated H_2_0 (Ambion) at 94°C for 90 s. The reaction was then incubated on ice for 2 min before reverse transcription was carried out in 20 µl total volume in the presence of 1 mM dNTPs, 10 U RNAsin, 200 U MMLV RTase (Moloney Murine Leukaemia Virus reverse transcriptase) and 1^st^ strand buffer (Invitrogen). Cycling conditions were 23°C 10 min, 37°C 40 min and 94°C 5 min before cDNA was diluted 12.5-fold in nuclease-free water. Quantitative real-time RT-PCR was carried out for each gene using the Opticon 2 real-time PCR machine (MJ Research) in a 25 µl reaction containing 400 nM primers, 200 nM probe and QuantiTect Probe PCR mix (Qiagen). Primer and probe sequences were as follows: forward 5′-CTG AGC AAA GAC CCC CAA CGA-3′, reverse 5′-GGC GGC GGT CAC GAA-3′ and probe 6-FAM-CGC GAT CAC ATG GTC CTG CTG G-TAMRA. Cycling conditions were: 50°C 2 min, 95°C 15 min, 39× (94°C 15 s, 60°C 60 s). Each sample was amplified in triplicate and mRNA estimation was made by comparison to duplicate standard curves and normalisation to β-actin [9].

### Native GFP imaging, immunohistochemistry and microscopy

For native GFP imaging, mice were transcardially perfused with 4% PFA after wash-through with PBS. Brains were carefully dissected and post-fixed in 4% PFA at 4°C overnight, then transferred to 30% sucrose in PBS and stored at 4°C overnight. Brains were embedded in OCT Compound (Tissue-Tek) and stored at −80°C until required. Coronal sections were cut to a thickness of 15 µm using a cryostat and delicately placed on Polysine slides (VWR international) in darkness and stored at −80°C until required. Sections were stained with the nuclear dye TOPRO-3 (Invitrogen, 1∶1000) and viewed on a Zeiss LSM510 confocal microscope. For immunofluorescence, mice were sacrificed by cervical dislocation and brains were quickly removed, frozen in isopentane on dry ice and stored at −80°C until required. Coronal sections were cut to a thickness of 15 µm using a cryostat (Bright Instrument Co. Ltd, UK), delicately placed on Polysine slides and stored at −80°C until required. Sections were fixed in 4% paraformaldehyde (PFA) for 30 min and washed in two changes of dH_2_0 before blocking for 15 min with 2% BSA in PBS. Sections were incubated with TOPRO-3, anti-GFP (Novus Biologicals, 1∶1000) and/or S830 anti-htt [39] (1∶2000) primary antibodies at 4°C overnight in darkness and washed in PBS for 15 min with gentle shaking. Fluorescent secondary antibodies Alexa-488-conjugated anti-rabbit (Molecular Probes, 1∶1000) and Alexa-555-conjugated anti-sheep (Molecular Probes, 1∶1000) were then incubated with the sections for 1 h at room temperature in darkness, followed by a 15 min wash in PBS with gentle shaking. Antibodies were diluted in 2% BSA in PBS. Sections were mounted with Mowiol-488 (Calbiochem) and viewed on a Zeiss LSM150 confocal microscope. For native and immunofluorescence studies, optimal confocal settings were obtained to limit background fluorescence and ensure detected fluorescent signals were not saturated, and remained the same throughout the experiment. Serial sections for native fluorescence detection were taken from four brains per genotype and for immunofluorescence studies from five brains per genotype. To quantify immunofluorescence of GFP-stained brain sections, fluorescent intensity measurements were taken of a fixed area between sections and within images using software supplied with the LSM510 confocal microscope. Once a fixed area had been established, areas of brain images were selected at random from different sections from different brains and the fluorescent intensity value was recorded. For quantification of fluorescence within nuclei, the size of the area chosen was enough to closely accommodate nuclei. Student's t-test was used to compare immunofluorescent intensity.
